# Pyrrolyl Pyrazoline Carbaldehydes as Enoyl-ACP Reductase Inhibitors: Design, Synthesis and Antitubercular Activity

**DOI:** 10.2174/1874104501711010092

**Published:** 2017-09-26

**Authors:** Sheshagiri R. Dixit, Shrinivas D. Joshi, Venkatarao H. Kulkarni, Sunil S. Jalalpure, Vijay M. Kumbar, Tulasigiriyappa Y. Mudaraddi, Mallikarjuna N. Nadagouda, Tejraj M. Aminabhavi

**Affiliations:** 1 Novel Drug Design and Discovery Laboratory, Department of Pharmaceutical Chemistry, Soniya Education Trust’s College of Pharmacy, Sangolli Rayanna Nagar, Dharwad-580 002, India; 2 KLE University’s College of Pharmacy, Nehru Nagar, Belagavi - 590010, Karnataka, India; 3 Basic Science Research Centre, KLE University, Belagavi - 590010, Karnataka, India

**Keywords:** Surflex docking, Cytotoxicity, Antitubercular activity, Pyrazoline carbaldehyde

## Abstract

**Introduction:**

In efforts to develop new antitubercular (anti-TB) compounds, herein we describe cytotoxic evaluation of 15 newly synthesized pyrrolyl pyrazoline carbaldehydes.

**Method & Materials:**

Surflex-Docking method was used to study binding modes of the compounds at the active site of the enzyme enoyl ACP reductase from *Mycobacterium tuberculosis (M. tuberculosis)*, which plays an important role in FAS-II biosynthetic pathway of *M. tuberculosis* and also it is an important target for designing novel anti-TB agents.

**Results:**

Among the synthesized compounds, compounds **4g** and **4i** showed H-bonding interactions with MET98, TYR158 and co-factor NAD^+^, all of which fitted well within the binding pocket of InhA. Also, these compounds have shown the same type of interaction as that of 4TZK ligand. The compounds were further evaluated for preliminary anti-TB activities against *M. tuberculosis* H37Rv strain.

**Conclusion:**

Some compounds were also screened for their mammalian cell toxicity using human lung cancer cell-line (A549) that was found to be nontoxic.

## 1 INTRODUCTION

Innovations on novel drugs for the treatment of tuberculosis (TB) have generated novel concepts in the field of medicinal chemistry, particularly keeping in mind on multi-drug resistance and high mortality rate [1, 2]. Presence of Human Immunodeficiency Virus (HIV), leading to Acquired Immune Deficiency Syndrome (AIDS) has provoked the occurrence of TB, which in turn caused the deaths among HIV-infected patients caused by co-infection with *Mycobacetrium tuberculosis* (*M. tuberculosis)*, thereby increasing the diminution of immune system [3]. There are several drugs in the market, which are currently in use, such as isoniazid (INH), rifampicin, pyrazinamide and delamanid as these possess a nitrogen atom in their ring structures *viz*., pyridine, piperazine, piperidine and pyrazine with azomethine and aryloxy moieties, which constitute their core structural features.

The association of multifunctional FAS-I system (fatty acid synthase type I) originate from eukaryotes provides a good opportunity for its selective inhibition, but it is relatively different from the individual enzymes of the bacterial fatty acid synthase type II system (FAS-II). Of the striking antimicrobial drug targets, FAS-II pathway has gained much attention in the modern era for developing INH based new chemical entities [4-7]. NADH-dependent enoyl acyl carrier protein (ENR) reductase encoded by *Mycobacterium* gene InhA is the key catalyst involved in mycolic acid biosynthesis in FAS-II patway. Earlier reports have recognized that InhA is the primary target for INH [8], a leading drug used for the treatment of TB for over 40 years. Reports have suggested that the formed INH-NADH adduct by the action of KatG (catalase-peroxide enzyme) on INH to form an acyl radical, which in turn covalently binds to NADH, acts as an effective inhibitor of InhA and other InhA inhibitors such as triclosan, pyrazole derivatives, diazaborines and indole-5-amides [9-13].

Chalcones have been studied extensively for their wide variety of pharmacological activities as antifungal [14], antimitotic [15], antitubercular [16], antitumor and antioxidant [17], antimalarial [18] and pyrazolines as these have shown a wide range of biological activities as anti-inflammatory [19, 20], antitumor [21], antitubercular [16], and monoamino oxidase inhibitor [22]. Pyrrole is also a vital part of the plant and animal origin as a hemin and vitamin B_12_ in animal cells, subunit of chlorophyll in plants. Earlier, pyrrole derivatives have shown *in vitro* anti-TB activity [23, 24], but recently, much research has occurred on anti-TB drug designing using pyrroles as the core structure in the synthesis and *in silico* studies [25-29], which suggests the significance of pyrrole derivatives as anti-TB agents. This has encouraged us to carry out a detailed study on the design and synthesis of new pyrrole derivatives as useful anti-TB agents.

In our earlier reports [16, 25], we have designed several inhibitors of InhA using fundamental core as a pyrrole ring with different pharmacophores in a single molecular skeleton along with Two- and Three-dimensional QSAR studies. Herein, we report the synthesis of pyrrole derivatives, which act as inhibitors of ENR enzyme along with their *in vitro* anti-TB studies. Some of the drugs that are under clinical or preclinical trials that are considered to synthesize new derivatives following the *Pall-Knorr* pyrrole synthesis are depicted in Fig. (**1**). Molecular docking studies have been employed to show the correlation between *in silico* results and *in vitro* analysis to find ENR as a prospective target of pyrrolyl-pyrazoline carbaldehyde derivatives.

## 2 MATERIALS AND METHODS

### 2.1 General Information

Melting points of the synthesized compounds were determined using digital melting point apparatus (Shital, Mumbai) and are uncorrected. FTIR spectra were recorded on a Bruker FTIR spectrophotometer using KBr pellets. The ^1^H and ^13^C NMR spectra were recorded on a Bruker AVANCE II at 400 and 100/75 MHz, respectively; chemical shifts are expressed in parts per million (δ ppm) relative to tetramethylsilane. Abbreviations used to describe the peak patterns are: (b) broad, (s) singlet, (d) doublet, (t) triplet, and (m) multiplet.

Mass spectra (MS) were recorded in a JEOL GCMATE II GC-Mass spectrometer and Schimadzu QP 20105 GC-Mass spectrometer. Analytical thin-layer chromatography (TLC) was performed on precoated TLC sheets of silica gel 60 F_254_ (Merck, Darmstadt, Germany) visualized by long- and short-wavelength UV lamps. Chromatographic purifications were performed on Merck silica gel (70-230 mesh).

### 2.2 General Procedure for the Synthesis of 1-(4-(1*H*-Pyrrol-1-yl) Phenyl)-3-Phenylprop-2-en-1-one (3a)

To a stirred alcoholic solution of pyrrolyl acetophenone **1** (1.57g, 0.01 mol), an alcoholic solution of substituted aldehydes **2(a-o)** (0.01 mol) and 40% sodium hydroxide (8 mL) were added with continuous stirring for 24-30 h. The resultant mixture was poured onto crushed ice and neutralized with hydrochloric acid. The separated solid was filtered, washed with water, dried and purified by column chromatography on a silica gel with ethyl acetate/petroleum ether (6:4) mixture as the eluent. Similar procedure was used to synthesize compounds (**3b-o**) [16].

### 2.3 General Procedure for the Synthesis of 3-(4-(1*H*-Pyrrol-1-yl) Phenyl)-5-Phenyl-4,5-Dihydro-1*H*-Pyrazole-1-Carbaldehydes (4a)

A mixture of chalcones (**3a**) (0.01 mmol), hydrazine hydrate 99% (0.01 mmol) and formic acid (5 mL) was refluxed for 18 h to complete the reaction as confirmed by TLC. Then the reaction mixture was cooled to ambient temperature; the separated solid was filtered, washed with cold ethanol, dried and purified by column chromatography on a silica gel with ethyl acetate/petroleum ether (6:4) mixture as the eluent to afford the compounds in high purity with good yields. The same procedure was used to synthesize compounds (**4b-o**).

(Yield 85%). mp 178-180^o^C; FTIR (KBr): υ, cm^-1^ 3138, 3029 (Ar-H), 1688 (C=O), 1607 (C=N); ^1^H NMR (400 MHz, CDCl_3_): δ, ppm: 3.28 (dd, J=4.88, 4.84 Hz, 1H, pyrazoline-C_4_-H_a_), 3.88 (dd, J=11.80, 11.76 Hz, 1H, pyrazoline-C_4_-H_b_), 5.60 (dd, J=4.80 Hz, 1H, pyrazoline-C_5_-H_x_), 6.41 (t, 2H, pyrrole-C_3_, C_4_-H), 7.17 (t, 2H, pyrrole-C_2_, C_5_-H), 7.28-7.49 (m, 7H, bridging phenyl-C_2_, C_6_ and ph-C_2_, C_3_, C_4_, C_5_, C_6_-H), 7.83 (t, 2H, bridging phenyl-C_3_, C_5_-H), 9.00 (d, J = 0.72 Hz, 1H, -CHO); ^13^C NMR (100 MHz, CDCl_3_): δ, ppm: 159.87, 154.62, 142.17, 133.60, 129.48, 128.66, 127.97, 120.14, 111.25, 55.71, 39.26; MS (EI): *m/z* = found 315 [M^+^]; calcd. 315.14.

#### 2.3.1 (4-(1H-Pyrrol-1-yl) Phenyl)-5-(4-Chlorophenyl)-4,5-Dihydro-1H-Pyrazole-1-Carbaldehyde (4b)

(Yield 70%). mp 168-170^o^C; FTIR (KBr): υ, cm^-1^ 3138, 3030 (Ar-H), 1667 (C=O), 1527 (C=N); ^1^H NMR (400 MHz, CDCl_3_): δ, ppm: 3.20 (dd, J=4.96, 5.00 Hz, 1H, pyrazoline-C_4_-H_a_), 3.84 (dd, J=11.80, 11.84 Hz, 1H, pyrazoline-C_4_-H_b_), 5.53 (dd, J=4.92 Hz, 1H, pyrazoline-C_5_-H_x_), 6.38 (t, 2H, pyrrole-C_3_, C_4_-H), 7.14 (t, 2H, pyrrole-C_2_, C_5_-H), 7.19 (d, J=1.72 Hz, 2H, chloroph-C_3_, C_5_-H), 7.30 (t, 2H, chloroph-C_2_, C_6_-H), 7.46 (d, J=8.68 Hz, 2H, bridging phenyl-C_2_, C_6_-H), 7.78 (d, J=1.68 Hz, 2H, bridging phenyl-C_3_, C_5_-H), 8.94 (d, J=0.36 Hz, 1H, -CHO); ^13^C NMR (100 MHz, CDCl_3_): δ, ppm: 160.01, 142.06, 132.06, 129.06, 128.03, 125.67, 120.08, 111.29, 59.14, 42.61; MS (EI): *m/z* = found 349 [M^+^]; calcd. 349.10.

#### 2.3.2 (4-(1H-pyrrol-1-yl)phenyl)-5-(2,3-Dichlorophenyl)-4,5-Dihydro-1H-Pyrazole-1-Carbaldehyde (4c)

(Yield 70%). mp 163-165^o^C; FTIR (KBr): υ, cm^-1^ 3108, 3066 (Ar-H), 1684 (C=O), 1528 (C=N); ^1^H NMR (400 MHz, CDCl_3_): δ, ppm: 3.12 (dd, J=5.08, 5.12 Hz, 1H, pyrazoline-C_4_-H_a_), 3.97 (dd, J=11.80 Hz, 1H, pyrazoline-C_4_-H_b_), 5.92 (dd, J=5.08 Hz, 1H, pyrazoline-C_5_-H_x_), 6.38 (t, 2H, pyrrole-C_3_, C_4_-H), 7.06 (dd, 1H, chlorophenyl-C_6_-H), 7.13 (t, 2H, pyrrole-C_2_, C_5_-H), 7.21 (t, 1H, chloroph-C_5_-H), 7.40-7.45 (m, 3H, chloroph-C_4_-H and bridging phenyl-C_2_, C_6_-H), 7.75-7.78 (m, 2H, bridging phenyl-C_3_, C_5_-H), 9.04 (d, J=0.36 Hz, 1H, -CHO); ^13^C NMR (100 MHz, CDCl_3_): δ, ppm: 160, 155.17, 142.34, 133.95, 129.90, 128.15, 127.85, 127.63, 120.01, 111.37, 57.32, 41.57.

#### 2.3.3 (4-(1H-pyrrol-1-yl)Phenyl)-5-(2,4-Dichlorophenyl)-4,5-Dihydro-1H-Pyrazole-1-Carbaldehyde (4d)

(Yield 75%). mp 228-230^o^C; FTIR (KBr): υ, cm^-1^ 3080, 3050 (Ar-H), 1665 (C=O), 1525 (C=N); ^1^H NMR (400 MHz, CDCl_3_) δ ppm: 3.11 (dd, J=5.24, 5.20 Hz, 1H, pyrazoline-C_4_-H_a_), 3.94 (dd, J=11.80, 11.84 Hz, 1H, pyrazoline-C_4_-H_b_), 5.85 (dd, J=5.16, 5.24 Hz, 1H, pyrazoline-C_5_-H_x_), 6.38 (t, 2H, pyrrole-C_3_, C_4_-H), 7.08 (d, J=8.40 Hz, 1H, chloroph-C_6_-H), 7.14 (t, 2H, pyrrole-C_2_, C_5_-H), 7.2-7.25 (m, 1H, chloroph-C_5_-H), 7.43-7.45 (m, 3H, chloroph-C_3_-H and bridging phenyl-C_2_, C_6_-H), 7.75-7.78 (m, 2H, bridging phenyl-C_3_, C_5_-H), 9.02 (s, 1H, -CHO); MS (EI): *m/z* = found 383 [M^+^]; calcd. 383.06.

#### 2.3.4 (4-(1H-pyrrol-1-yl)Phenyl)-5-(2,6-Dichlorophenyl)-4,5-Dihydro-1H-Pyrazole-1-Carbaldehyde (4e)

(Yield 70%). mp 208-210^o^C; FTIR (KBr): υ, cm^-1^ 3118, 3099 (Ar-H), 1667 (C=O), 1525 (C=N); ^1^H NMR (400 MHz, CDCl_3_) δ ppm: 3.40 (dd, J=8.32 Hz, 1H, pyrazoline-C_4_-H_a_), 3.78 (dd, J=12.84 Hz, 1H, pyrazoline-C_4_-H_b_), 6.21-6.26 (m, 1H, pyrazoline-C_5_-H_x_), 6.39 (t, 2H, pyrrole-C_3_, C_4_-H), 7.15 (t, 2H, pyrrole-C_2_, C_5_-H), 7.20 (d, J=8.04 Hz, 1H, chloroph-C_4_-H), 7.28-7.48 (m, 4H, chloroph-C_3_, C_5_-H and bridging phenyl-C_2_, C_6_-H), 7.78-7.82 (m, 2H, bridging phenyl-C_3_, C_5_-H), 8.89 (d, J=1.04 Hz, 1H, -CHO); ^13^C NMR (100 MHz, CDCl_3_): δ, ppm: 159.99, 154.67, 142.30, 139.05, 134.26, 129.26, 128.12, 127.20, 120.08, 111.35, 58.56, 42.45.

#### 2.3.5 (4-(1H-pyrrol-1-yl)Phenyl)-5-(p-Tolyl)-4,5-Dihydro-1H-Pyrazole-1-Carbaldehyde (4f)

(Yield 60%). mp 148-150^o^C; FTIR (KBr): υ, cm^-1^ 3142, 3051 (Ar-H), 1686 (C=O), 1527 (C=N); ^1^H NMR (400 MHz, CDCl_3_): δ, ppm: 3.23 (dd, J=4.84 Hz, 1H, pyrazoline-C_4_-H_a_), 3.83 (dd, J=11.76 Hz, 1H, pyrazoline-C_4_-H_b_), 5.54 (dd, J=5.00, 4.68 Hz, 1H, pyrazoline-C_5_-H_x_), 6.38 (t, 2H, pyrrole-C_3_, C_4_-H), 7.13-7.16 (m, 6H, pyrrole-C_2_, C_5_-H and methylph-C_2_, C_3_, C_5_, C_6_-H), 7.43-7.46 (m, 2H, bridging phenyl-C_2_, C_6_-H), 7.77-7.80 (m, 2H, bridging phenyl-C_3_, C_5_-H), 8.95 (d, J=0.68 Hz, 1H, -CHO); ^13^C NMR (100 MHz, CDCl_3_): δ, ppm: 160.24, 155.01, 142.49, 142.26, 131.19, 130.43, 129.96, 128.17, 124.82, 120.21, 111.33, 64.91, 42.61, 29.70; MS (EI): *m/z* = found 329 [M^+^]; calcd. 329.40.

#### 2.3.6 (4-(1H-pyrrol-1-yl)phenyl)-5-(4-Methoxyphenyl)-4,5-Dihydro-1H-Pyrazole-1-Carbaldehyde (4g)

(Yield 70%). mp 112-114^o^C; FTIR (KBr): υ, cm^-1^ 3136, 3075 (Ar-H), 1671 (C=O), 1529 (C=N); ^1^H NMR (400 MHz, CDCl_3_): δ, ppm: 3.24 (dd, J=4.84 Hz, 1H, pyrazoline-C_4_-H_a_), 3.74-3.82 (m, 4H, pyrazoline-C_4_-H_b_ and -OCH_3_), 5.52 (dd, J=4.76, 4.72 Hz, 1H, pyrazoline-C_5_-H_x_), 6.38 (t, 2H, pyrrole-C_3_, C_4_-H), 6.84-6.88 (m, 2H, methoxyph-C_3_, C_5_-H), 7.14 (t, 2H, pyrrole-C_2_, C_5_-H), 7.17-7.21 (m, 2H, methoxyph-C_2_, C_6_-H), 7.43-7.46 (m, 2H, bridging phenyl-C_2_, C_6_-H), 7.77-7.80 (m, 2H, bridging phenyl-C_3_, C_5_-H), 8.94 (d, J=0.92 Hz, 1H, -CHO); ^13^C NMR (100 MHz, CDCl_3_): δ, ppm: 160.01, 159.35, 154.83, 142.16, 132.79, 128.10, 127.06, 120.05, 114.43, 111.30, 58.69, 55.30, 42.54; MS (EI): *m/z* = found 345 [M^+^]; calcd. 345.15.

#### 2.3.7 (4-(1H-Pyrrol-1-yl)Phenyl)-5-(3-Methoxyphenyl)-4,5-Dihydro-1H-Pyrazole-1-Carbaldehyde (4h)

(Yield 70%). mp 158-160^o^C; FTIR (KBr): υ, cm^-1^ 3131, 3073 (Ar-H), 1668 (C=O), 1527 (C=N); ^1^H NMR (400 MHz, CDCl_3_) δ ppm: 3.24 (dd, J=4.84, 4.88 Hz, 1H, pyrazoline-C_4_-H_a_), 3.77-3.82 (m, 4H, pyrazoline-C_4_-H_b_ and -OCH_3_), 5.54 (dd, J=4.80, 4.76 Hz, 1H, pyrazoline-C_5_-H_x_), 6.39 (t, 2H, pyrrole-C_3_, C_4_-H), 6.78-6.86 (m, 3H, methoxyph-C_2_, C_4_, C_6_-H), 7.14 (t, 2H, pyrrole-C_2_, C_5_-H), 7.26 (t, 1H, methoxyph-C_5_-H), 7.43-7.46 (m, 2H, bridging phenyl-C_2_, C_6_-H), 7.76-7.80 (m, 2H, bridging phenyl-C_3_, C_5_-H), 8.98 (d, J=0.92 Hz, 1H, -CHO); ^13^C NMR (100 MHz, CDCl_3_): δ, ppm: 160.03, 154.79, 142.16, 130.20, 128.10, 120.07, 119, 113.18, 111.28, 59.06, 55.26, 42.63; MS (EI): *m/z* = found 345 [M^+^]; calcd. 345.15.

#### 2.3.8 (4-(1H-Pyrrol-1-yl)Phenyl)-5-(2,4-Dimethoxyphenyl)-4,5-Dihydro-1H-Pyrazole-1-Carbaldehyde (4i)

(Yield 70%). mp 160-162^o^C; FTIR (KBr): υ, cm^-1^ 3106, 3011 (Ar-H), 1671 (C=O), 1527 (C=N); ^1^H NMR (400 MHz, CDCl_3_): δ, ppm: 3.13 (dd, J=4.92 Hz, 1H, pyrazoline-C_4_-H_a_), 3.69-3.82 (m, 7H, pyrazoline-C_4_-H_b_ and -2OCH_3_), 5.73 (dd, J=4.88, 4.80 Hz, 1H, pyrazoline-C_5_-H_x_), 6.38 (t, 2H, pyrrole-C_3_, C_4_-H), 6.45 (dd, J=2.32, 2.52 Hz, 1H, methoxyph-C_3_-H), 6.48 (d, J=2.32 Hz, 1H, methoxyph-C_5_-H), 7.03 (d, J=8.36 Hz, 1H, methoxyph-C_6_-H), 7.14 (t, 2H, pyrrole-C_2_, C_5_-H), 7.41-7.45 (m, 2H, bridging phenyl-C_2_, C_6_-H), 7.76-7.79 (m, 2H, bridging phenyl-C_3_, C_5_-H), 8.99 (d, J=0.80 Hz, 1H, -CHO); ^13^C NMR (100 MHz, CDCl_3_): δ, ppm: 160.67, 160.04, 157.27, 155.67, 141.45, 128.45, 127.12, 120.43, 119.01, 111.19, 104.24, 99.22, 55.49, 55.40, 55.10, 41.68.

#### 2.3.9 (4-(1H-Pyrrol-1-yl) Phenyl)-5-(3,4-Dimethoxyphenyl)-4,5-Dihydro-1H-Pyrazole-1-Carbaldehyde (4j)

(Yield 70%). mp 133-135^o^C; FTIR (KBr): υ, cm^-1^ 3141, 3101 (Ar-H), 1679 (C=O), 1521 (C=N); ^1^H NMR (400 MHz, CDCl_3_) δ ppm: 3.26 (dd, J=4.92 Hz, 1H, pyrazoline-C_4_-H_a_), 3.76-3.98 (m, 7H, pyrazoline-C_4_-H_b_ and -2OCH_3_), 5.53 (dd, J=4.88, 3.96 Hz, 1H, pyrazoline-C_5_-H_x_), 6.39 (t, 2H, pyrrole-C_3_, C_4_-H), 6.77 (s, 1H, methoxyph-C_6_-H), 6.82 (s, 2H, methoxyph-C_2_, C_5_-H), 7.15 (t, 2H, pyrrole-C_2_, C_5_-H), 7.45-7.47 (m, 2H, bridging phenyl-C_2_, C_6_-H), 7.79-7.81 (m, 2H, bridging phenyl-C_3_, C_5_-H), 8.97 (d, J=0.88 Hz, 1H, -CHO); ^13^C NMR (100 MHz, CDCl_3_): δ, ppm: 160.08, 154.86, 148.90, 142.21, 132.20, 128.10, 120.08, 118.99, 117.88, 111.67, 109.11, 58.97, 56, 55.97, 42.64; MS (EI): *m/z* = found 375 [M^+^]; calcd. 375.16.

#### 2.3.10 (4-(1H-Pyrrol-1-yl)phenyl)-5-(4-Fluorophenyl)-4,5-Dihydro-1H-Pyrazole-1-Carbaldehyde (4k)

(Yield 70%). mp 123-125^o^C; FTIR (KBr): υ, cm^-1^ 3144, 3070 (Ar-H), 1664 (C=O), 1526 (C=N); ^1^H NMR (400 MHz, CDCl_3_): δ, ppm: 3.22 (dd, J=4.92, 4.88 Hz, 1H, pyrazoline-C_4_-H_a_), 3.85 (dd, J=11.80 Hz, 2H, pyrazoline-C_4_-H_b_), 5.55 (dd, J=4.80, 4.84 Hz, 1H, pyrazoline-C_5_-H_x_), 6.40 (dd, J=2.20, 3.24 Hz, 2H, pyrrole-C_3_, C_4_-H), 7.01-7.06 (m, 2H, fluoroph-C_3_, C_5_-H), 7.13-7.16 (m, 2H, pyrrole-C_2_, C_5_-H), 7.22-7.26 (m, 2H, fluoroph-C_2_, C_6_-H), 7.44-7.47 (m, 2H, bridging phenyl-C_2_, C_6_-H), 7.77-7.80 (m, 2H, bridging phenyl-C_3_, C_5_-H), 8.95 (d, J=0.72 Hz, 1H, -CHO); ^13^C NMR (100 MHz, CDCl_3_); δ, ppm: 163.96, 160.77, 154.74, 142.28, 136.43, 128.12, 127.85, 120.07, 115.82, 111.34, 58.50, 42.54; MS (EI): *m/z* = found 313 [M^+^]; calcd. 313.13.

#### 2.3.11 (4-(1H-Pyrrol-1-yl)Phenyl)-5-(4-Isopropylphenyl)-4,5-Dihydro-1H-Pyrazole-1-Carbaldehyde (4l)

(Yield 70%). mp 140-142^o^C; FTIR (KBr): υ, cm^-1^ 3106, 2923 (Ar-H), 1672 (C=O), 1526 (C=N); ^1^H NMR (400 MHz, CDCl_3_): δ, ppm: 1.25 (s, 1H, -CH(CH_3_)_2_), 3.12 (dd, J=4.92, 1H, pyrazoline-C_4_-H_a_), 3.68-3.83 (m, 7H, pyrazoline-C_4_-H_b_ and –CH(CH_3_)_2_), 5.73 (dd, J=4.84, 1H, pyrazoline-C_5_-H_x_), 6.38 (dd, J=2.24, 2.76 Hz, 2H, pyrrole-C_3_, C_4_-H), 6.41-6.47 (m, 2H, isopropylph-C_3_, C_5_-H), 7.00-7.19 (m, 4H, pyrrole-C_2_, C_5_-H and isopropylph-C_2_, C_6_-H), 7.41-7.45 (m, 2H, bridging phenyl-C_2_, C_6_-H), 7.76-7.79 (m, 2H, bridging phenyl-C_3_, C_5_-H), 8.99 (d, J=0.92 Hz, 1H, -CHO); ^13^C NMR (100 MHz, CDCl_3_); δ, ppm: 160.06, 155.72, 142.01, 128.43, 127.13, 125.64, 120.43, 111.28, 58.92, 42.58, 33.78, 23.88; MS (EI): *m/z* = found 357 [M^+^]; calcd. 357.18.

#### 2.3.12 (4-(1H-Pyrrol-1-yl)Phenyl)-5-(3-Bromophenyl)-4,5-Dihydro-1H-Pyrazole-1-Carbaldehyde (4m)

(Yield 75%). mp 168-170^o^C; FTIR (KBr): υ, cm^-1^ 3138, 3100 (Ar-H), 1670 (C=O), 1524 (C=N); ^1^H NMR (400 MHz, CDCl_3_): δ, ppm: 3.21 (dd, J=5.00 Hz, 1H, pyrazoline-C_4_-H_a_), 3.84 (dd, J=11.84 Hz, 1H, pyrazoline-C_4_-H_b_), 5.52 (dd, J=4.96, 4.80 Hz, 1H, pyrazoline-C_5_-H_x_), 6.38 (t, 2H, pyrrole-C_3_, C_4_-H), 7.13-8.12 (m, 10H, pyrrole-C_2_, C_5_, bromoph-C_2_, C_4_, C_5_, C_6_-H and bridging phenyl-C_2_, C_3,_ C_5_, C_6_-H), 8.96 (d, J=0.88 Hz, 1H, -CHO); ^13^C NMR (100 MHz, CDCl_3_): δ, ppm: 160.04, 154.73, 142.93, 142.31, 133.29, 131.24, 128.76, 124.44, 123.16, 120.06, 111.71, 58.56, 42.50.

#### 2.3.13 (4-(1H-pyrrol-1-yl)Phenyl)-5-(3-Phenoxyphenyl)-4,5-Dihydro-1H-Pyrazole-1-Carbaldehyde (4n)

(Yield 78%). mp 128-130^o^C; FTIR (KBr): υ, cm^-1^ 3058 (Ar-H), 1678 (C=O), 1524 (C=N); ^1^H NMR (400 MHz, CDCl_3_) δ ppm: 3.23 (dd, J=4.84, 4.88 Hz, 1H, pyrazoline-C_4_-H_a_), 3.83 (dd, J=11.76 Hz, 1H, pyrazoline-C_4_-H_b_), 5.55 (dd, J=4.80, 4.76 Hz, 1H, pyrazoline-C_5_-H_x_), 6.38 (t, 2H, pyrrole-C_3_, C_4_-H), 6.93-7.83 (m, 15H, pyrrole-C_2_, C_5_, phenoxyph-C_2_, C_4_, C_5_, C_6_, C_7_, C_8_, C_9_, C_10_, C_11_-H and bridging phenyl-C_2_, C_3,_ C_5_, C_6_-H), 8.97 (d, J=0.84 Hz, 1H, -CHO); ^13^C NMR (100 MHz, CDCl_3_): δ, ppm: 160, 154.77, 142.16, 137.79, 129.71, 128.09, 125.64, 120.07, 119, 111.28, 58.96, 42.61.

#### 2.3.14 (4-(1H-Pyrrol-1-yl)phenyl)-5-(Furan-2-yl)-4,5-Dihydro-1H-pyrazole-1-Carbaldehyde (4o)

(Yield 80%). mp 152-154^o^C; FTIR (KBr): υ, cm^-1^ 3140, 3117 (Ar-H), 1670 (C=O), 1524 (C=N); ^1^H NMR (400 MHz, CDCl_3_): δ, ppm: 3.53 (dd, J=5.04 Hz, 1H, pyrazoline-C_4_-H_a_), 3.68 (dd, J=11.60 Hz, 1H, pyrazoline-C_4_-H_b_), 5.67 (dd, J=5.00 Hz, 1H, pyrazoline-C_5_-H_x_), 6.33-6.39 (m, 4H, pyrrole-C_3_, C_4_-H and furan-C_3_, C_4_-H), 7.14-7.17 (m, 2H, pyrrole-C_2_, C_5_-H) 7.33 (dd, J=0.64, 0.60 Hz, 1H, furan- C_5_-H), 7.44-7.47 (m, 2H, bridging phenyl-C_2_, C_6_-H), 7.78-7.82 (m, 2H, bridging phenyl-C_3_, C_5_-H), 8.90 (d, J=0.88 Hz, 1H, -CHO); ^13^C NMR (100 MHz, CDCl_3_): δ, ppm: 160.04, 154.97, 150.90, 142.36, 142.21, 128.12, 120.04, 111.36, 110.65, 108.17, 52.54, 38.47; MS (EI): *m/z* = found 305 [M^+^]; calcd. 305.12.

## 3 COMPUTATIONAL METHODS

Sybyl package was used to draw 3D structures (Tripos Associates, St. Louis, MO, USA) [30]. Tripose force field was used for geometric optimization [31] using a distance dependent-dielectric function, energy gradient of 0.001 kcal/mol and MMFF94 as the electrostatics. Repeated molecular dynamics-based simulated annealing approach was implemented for the conformational analyses of all the 15 compounds. Molecules were heated up to 1000 K within 2000 fs, held at this temperature for 2000 fs and annealed to 0 K for 10,000 fs using an exponential annealing function. By employing this procedure, 100 conformations were sampled out during the 100 cycles to account for conformational flexibility to find the most likely stable conformations. All the conformations were minimized with Tripos force field and atomic charges were calculated using MMFF94 (Merck Molecular Force Field) method.

### 3.1 Molecular Docking Using Surflex-Dock

Molecular docking was performed to elucidate the type of binding of the compounds to afford uncomplicated information for further structural optimization. Surflex-Dock that adopted an experimental scoring function and a patented searching engine [32, 33] was employed in molecular docking studies. The crystal structure of *M. tuberculosis* enoyl reductase (InhA) complexed with 1-cyclohexyl-*N*-(3,5-dichlorophenyl)-5-oxopyrrolidine-3-carboxamide (PDB ID 4TZK, 1.62 Å X-ray resolution) was downloaded from the Brookhaven Protein Database (PDB http://www.rcsb.org/pdb). During the preparation of protein for docking study, ligands and water molecules present in the crystal structure were removed (except co-factor NAD^+^), then the enzyme 4TZK was assigned with polar hydrogens and united atom Amber7 FF99. Then, ligand-based mode was used to produce the “protomol”, keeping the threshold and bloat parameters at their default values of 0.50 and 0 Å all the inhibitors were docked within the prepared protein.

Interaction mode of the ligand present in the crystal structure was used as a standard docked model against 4TZK PDB. The maximum of 20 numbers of maximum orientations per ligand was set with no constraints to perform molecular docking. Docking complex was believed to show the ligand-receptor interactions, which was selected based on three criteria: (i) docking score of the orientation possessed the highest docking score, (ii) orientation of aromatic rings of the ligand into the active site in a similar manner with the co-crystallized ligand orientations, and (iii) preservation of H-bonding interactions with Tyr158 and Co-factor NAD^+^. For comparative study of the designed molecules, the following parameters were estimated using the C-Score module of the Sybyl-X 2.0, D_score [34], PMF_score [35], G_score [36] and Chem_score [37].

## 4 BIOLOGICAL ACTIVITY

### 4.1 Antitubercular Activities

Microplate almar blue assay method was used to study the inhibition of *M. tuberculosis* H_37_Rv of all the synthesized compounds [38]. The 96 wells containing plate charged with 100 mL of Middlebrook 7H9 broth and serial dilution of compounds with the drug concentrations of 0.2, 0.4, 0.8, 1.6, 3.125, 6.25, 12.5, 25, 50 and 100 mg/mL were added directly on the plate, sealed with parafilm and incubated for 5 days at 37^o^ C. Then a mixture (1:1) of almar blue reagent and 10% Tween 80 (25 mL) were added to the plate and incubated for 24 h. Bacterial growth was interpreted by the development of pink color, while blue color suggests no bacterial growth. The MIC, defined as the lowest drug concentration, prevented the color change from blue to pink. Table **1** reveals anti-TB activity data expressed in MIC.

### 4.2 MTT-Based Cytotoxicity Activity

Cellular conversion of MTT [3-(4,5-dimethylthiazo-2-yl)-2,5-diphenyl-tetrazolium bromide] into a formazan product [39] was used to estimate cytotoxic activity (IC_50_) of some of the compounds against cell-line A549 (lung adenocarcinoma) up to concentrations of 100 µg/mL using 5×10^3^cells/well in a 96-well flat-bottom micro plate and maintained at 37^0^C in 95% humidity and 5% CO_2_ for overnight using paclitaxel as the positive control. The IC_50_ values are the averages (± SEM) of three independent experiments, which are presented in Table **2**.

## 5 RESULTS AND DISCUSSION

### 5.1 Chemistry

Synthesis of all the compounds was carried out as depicted in Scheme (**1**). The *Paal-Knorr* synthesis involved the reaction of diketone like 2,5-dimethoxy tetrahydrofuran with amine, which is among the most conventional method for the synthesis of heterocyclic pyrrole ring. Hence, (4-pyrrol-1-yl)acetophenone (**1**) was obtained by forming pyrrole ring using the amino group present at the *para-* position of aromatic ring of 4-amino acetophenone in the presence of dried glacial acetic acid. The required key intermediates *viz*., chalcones (**3a-o**) were synthesized using Claisen-Schmidt condensation reaction between (4-pyrrol-1-yl)acetophenone (**1**) and substituted aldehydes (**2a-o**) in the presence of 40% alcoholic sodium hydroxide. Thus, synthesized chalcones (**3a-o**) were then cyclized to pyrazolyl carbaldehyde (**4a-o**) in a solvent free atmosphere by treating with hydrazine hydrate and formic acid.

All the synthesized compounds were characterized by FTIR, ^1^H NMR, ^13^C NMR and mass spectroscopy. FTIR showed the presence of carbonyl and C=N groups as strong absorption bands in the regions 1664-1688 cm^-1^ and 1521-1607 cm^-1^, respectively. In ^1^H NMR spectra resonating signals of formyl group (-CHO), protons are observed as doublets in the range δ 8.89-9.04 ppm. The pyrazoline protons at the 4-H position appeared as two doublet of doublet/multiplet from δ 3.11-3.97 and that of protons at the 5-H position appeared as a doublet of doublet/triplet from δ 5.52-6.26. The pyrrole protons at the 3-H and 4-H positions appeared as a triplet from δ 6.38-6.41 ppm and those protons at the 2-H and 5-H positions resonated as triplet/multiplet from δ 7.06-7.17 ppm. The ^13^C NMR spectra exhibited characteristic signals of carbonyl carbon atom in the range of δ 159.87-163.96 ppm. The mass spectra showed a precise molecular ion peak data for the respective compounds.

#### 5.1.1 Antitubercular Activities

Anti-TB activity was determined for compounds **4(a-o)** against *M. tuberculosis* strain H37Rv by a microplate alamar blue assay (MABA) using pyrazinamide and streptomycin as reference drugs and these results are depicted in Table **1**. The initial anti-TB evaluation showed that majority of compounds exhibited moderate to good activity. The activities of compounds **4(a-o)** are expressed in terms of MIC values. Compound **4i** showed the highest activity with a MIC value of 3.125 µg/ml, while compounds **4g** and **4h** showed better activity with a MIC value of 6.25 µg/ml. A good anti-TB activity is due to the presence of pharmacologically active hetero-aryl pyrazoline ring with formyl group.

#### 5.1.2 MTT-Based Cytotoxicity Studies

Certain therapeutic properties are to be determined to examine the antimycobacterial potential of a drug. Toxicity is one of these criteria. Hence, we have investigated the potential toxicity of eight selected pyrrolyl derivatives (**4b, 4h, 4i, 4j, 4l, 4m** and **4n**) towards A549 (lung adenocarcinoma) cell-lines up to concentrations of 100 µg/mL. These compounds showed a moderate cytotoxicity compared to paclitaxel (see Table **2**). Specifically, the most potent compounds *viz*., **4j** and **4l** exhibited a good safety profile as their IC_50_ value were 70.36 and 71.07 µmol/L, respectively against A549 cell-line.

### 5.2 Molecular Docking Results

**Fig. (3):**
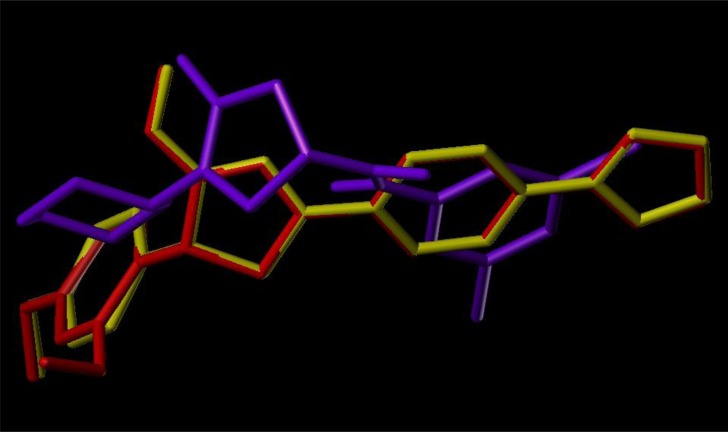
Orientation and superimposition of active analogs **4i** (red) and **4g** (yellow) over 4TZK crystal ligand (purple).

To examine the mechanism of anti-TB activity and to understand the comprehensive intermolecular interactions between the synthesized compounds and the enzyme, molecular docking studies were performed on the crystal structure of *M. tuberculosis* enoyl reductase (InhA) complexed with 1-cyclohexyl-*N*- (3,5-dichlorophenyl)-5-oxopyrrolidine-3-carboxamide (PDB ID 4TZK, 1.62 Å X-ray resolution) using surflex-dock programme of sybyl-X 2.0 software. Based on the greater level of resistance associated with INH isolates against InhA, docking studies were performed on InhA complex with 1-cyclohexyl-*N*-(3,5-dichlorophenyl)-5-oxopyrrolidine-3-carboxamide, which indicates the presence of drug-receptor interactions. All the 15 inhibitors were docked into the active site of ENR as shown in Figs. (**2A** and **B**). The predicted binding energies of the compounds are listed in Table (**3**). Superimposition of compounds **4i** and **4g** with ligand of 4TZK is depicted in Fig. **3**.

**Fig. (7):**
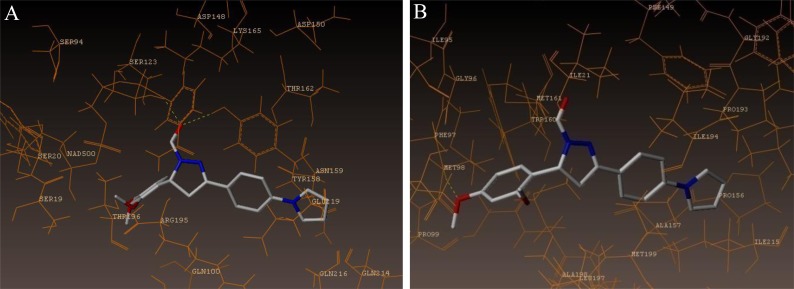
(A) Hydrophilic and (B) Hydrophobic amino acids surrounded to representative compound **4i.**

The interaction of 4TZK ligand with the enzyme depicted in Figs. (**4A**-**C**) shows that oxygen atom of carbonyl group present on pyrrolidine ring has two H-bonding interactions with the hydrogens of TYR158 and NAD^+^ (-C=O ------ H-TYR158, 1.84 Å; H-NAD^+^, 2.20 Å). As depicted in Figs. (**5A**-**C**), the oxygen atom of formyl group present at the 2^nd^ position of pyrazoline ring of compound **4i** exhibited two H-bonding interactions with the hydrogens of TYR158 and NAD^+^ (-CHO ------ H-TYR158, 2.22 Å; H-NAD^+^, 1.99 Å), while the oxygen atom of -OCH_3_ present at the 4^th^ position of aromatic ring makes H-bonding interactions with MET98 (-OCH_3_ ----- H-MET98, 1.98 Å). In Figs. (**6A**-**C**), the oxygen atom of formyl group present at the 2^nd^ position of pyrazoline ring of compound **4g** makes two H-bonding interaction with the hydrogens of TYR158 and NAD^+^ (-CHO ------ H-TYR158, 2.21 Å; H-NAD^+^, 2.01 Å), while the oxygen atom of -OCH_3_ present at the 4^th^ position of aromatic ring exhibited H-bonding interaction with MET98 (-OCH_3_ ----- H-MET98, 1.92 Å). On the other hand, hydrophilic (SER94, SER123, SER20, NAD500, SER19, THR196, ARG195, GLN100, GLN216, GLN214, GLU219, TYR158, ASN159, THR162, ASP150, LYS165, ASP148) and hydrophobic (GLY96, PHE97, MET98, PRO99, TRP160, MET161, ILE21, MET199, ALA157, PRO156, ILE215, ILE194, PRO193, PHE149,ALA198, LEU197) amino acid residues surrounded over the representative compound **4i** are represented in (Figs. **7A** and **B**).

All the compounds showed consensus scores in the range of 9.05-2.65, indicating the summary of all forces of interactions between ligands and InhA. Charge and van der Waals interactions between protein and ligands varied from -77.96 to -168.11. Helmholtz free energies of interactions for protein ligands atom pairs range between -33.56 and -66.28. However, its H-bonding, complex (ligand-protein), and internal (ligand-ligand) energies range from -151.65 to -285.29, while those values of -25.78 to -45.80 indicate the ligands due to H-bonding, lipophilic contact, and rotational entropy as well as intercept terms. These scores indicate that molecules preferentially bind to InhA in comparison to reference 4 TZK ligand (Table **3**). In general, it is observed that -OCH_3_ and CHO groups make the H-bond at substrate binding site and the presence of electron donating or withdrawing substitution on the aromatic ring attached to pyrazoline moiety may be helpful to show the activity, while those of pyrrole and pyrazoline moieties help to occupy or penetrate the molecule at the active sites.

The Lipinski’s ‘rule of 5’ [40] was calculated for compounds **4(a-o)** to evaluate their drug likeness property, which in general states that an orally active drug did not have more than one violation *viz*., (i) no more than five H-bond donors, (ii) molecular weight < 500 daltons, (iii) cLogP not > 5, and (iv) no more than 10 H-bond acceptors [38]. We have calculated theoretical cLogP, molecular weight (MW) and number of H-bond donors and acceptors using Sybyl-X.2.0. Examination of the results summerized in (Table **4**), suggests that compounds **4(a-o)** satisfied the range of physicochemical parameters established by the Lipinski’s rule.

## CONCLUSION

In this study, novel compounds 3-(4-(1*H*-pyrrol-1-yl)phenyl)-5-substituted phenyl-4,5-dihydro-1*H*-pyrazole-1-carbaldehydes **4(a-n)** and 3-(4-(1*H*-pyrrol-1-yl)phenyl)-5-(furan-2-yl)-4,5-dihydro-1*H*-pyrazole-1-carbaldehyde **(4o)** have been synthesized that were recognized as potent InhA inhibitors by *in silico* studies. These pyrrole derivatives were further explored in search of novel anti-TB agents, identifying several derivatives with the reasonable inhibitory activities against *M. tuberculosis*. Of all the compounds tested, compound **4i** exhibited significant activity with a MIC value of 3.125 µg/mL and compounds **4g** and **4h** have displayed the MIC value of 6.25 µg/mL with no apparent cytotoxicites towards human lung cancer cell-line (A549). This indicates non-toxic behaviour of chemophores and hence, they may be considered for further structural modification.

Molecular docking was performed to understand drug-receptor interactions. The results have shown that the compounds are mainly bound to the substrate binding site of InhA and the scoring function for most of the compounds is similar to that of the reference inhibitor. The anti-TB activity of these compounds was fully supported by *in silico* molecular docking calculations. The synthesized compounds were found to be useful as the lead compounds for further developing InhA inhibitors. Our future goal is to identify the mechanism of action and explore the pyrrole analogues, which might serve as a new template for further investigations amid selective and less toxic anti-TB agents to merit the cost effective and reduced treatment time. More studies are underway to improve their efficiency against InhA and *M. tuberculosis*.

## Figures and Tables

**Fig. (1):**
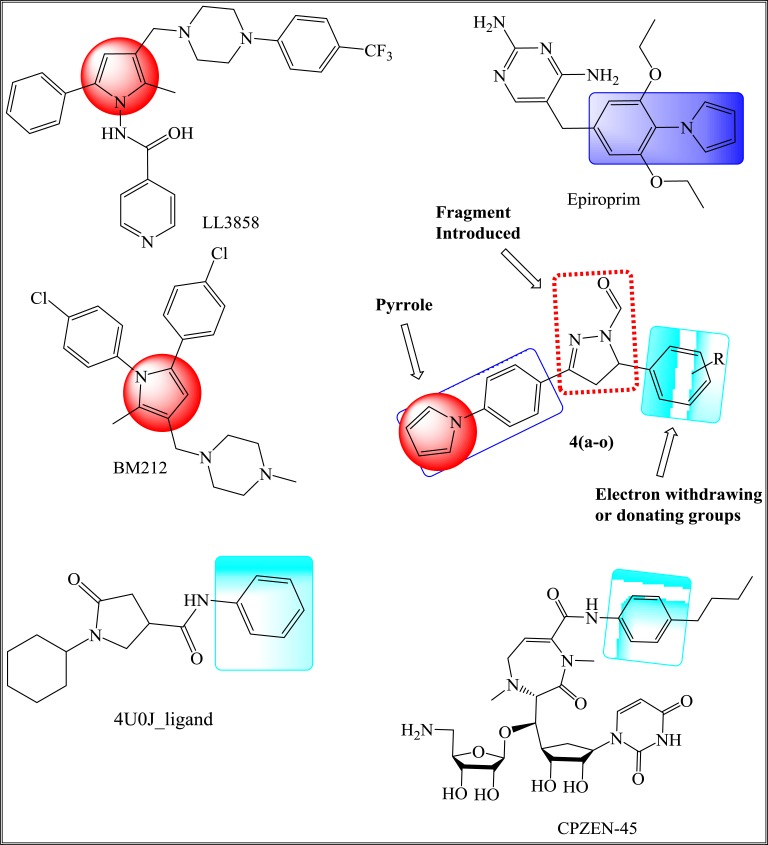
Design concept for the synthesis of titled compounds.

**Scheme 1:**
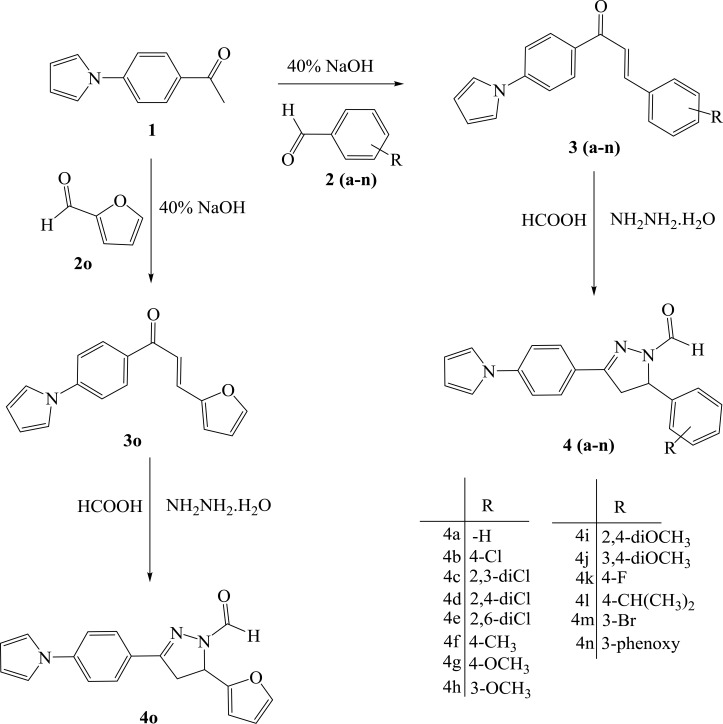
Synthetic route of novel series of pyrrolyl carbaldehyde derivatives.

**Fig. (2):**
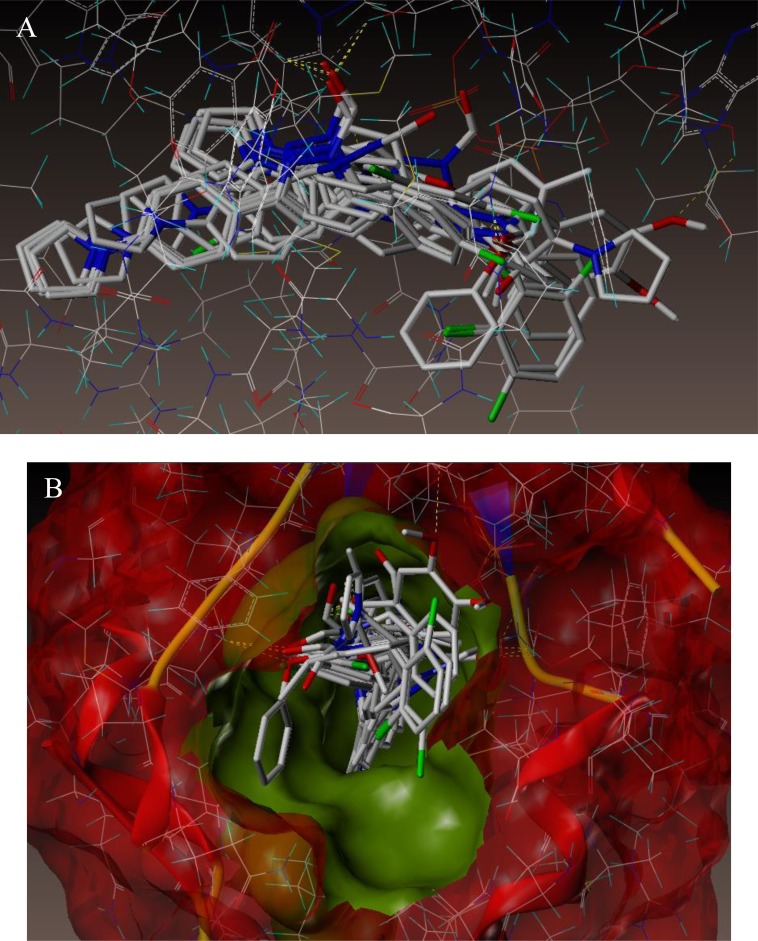
**(A)** Docked mode of all molecules; (B) Inside the proposed binding pocket of InhA.

**Fig. (4):**
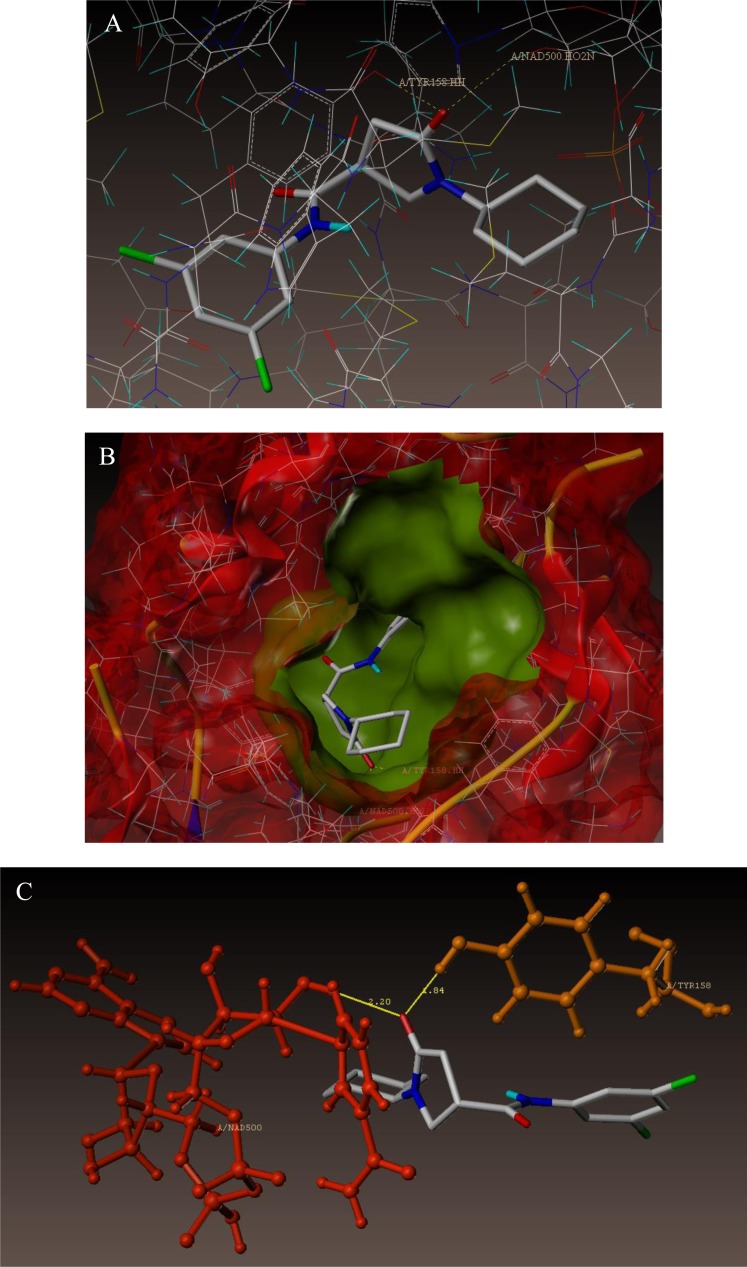
(A) Docked mode of **4TZK_ligand**; (B) Inside the proposed binding pocket of InhA; (C) 3D docked view of the **4TZK_ligand**. Binding site residues; red colored Try158 amino acid, blue colored co-factor NAD+ and the molecule is colored according to atom type.

**Fig. (5):**
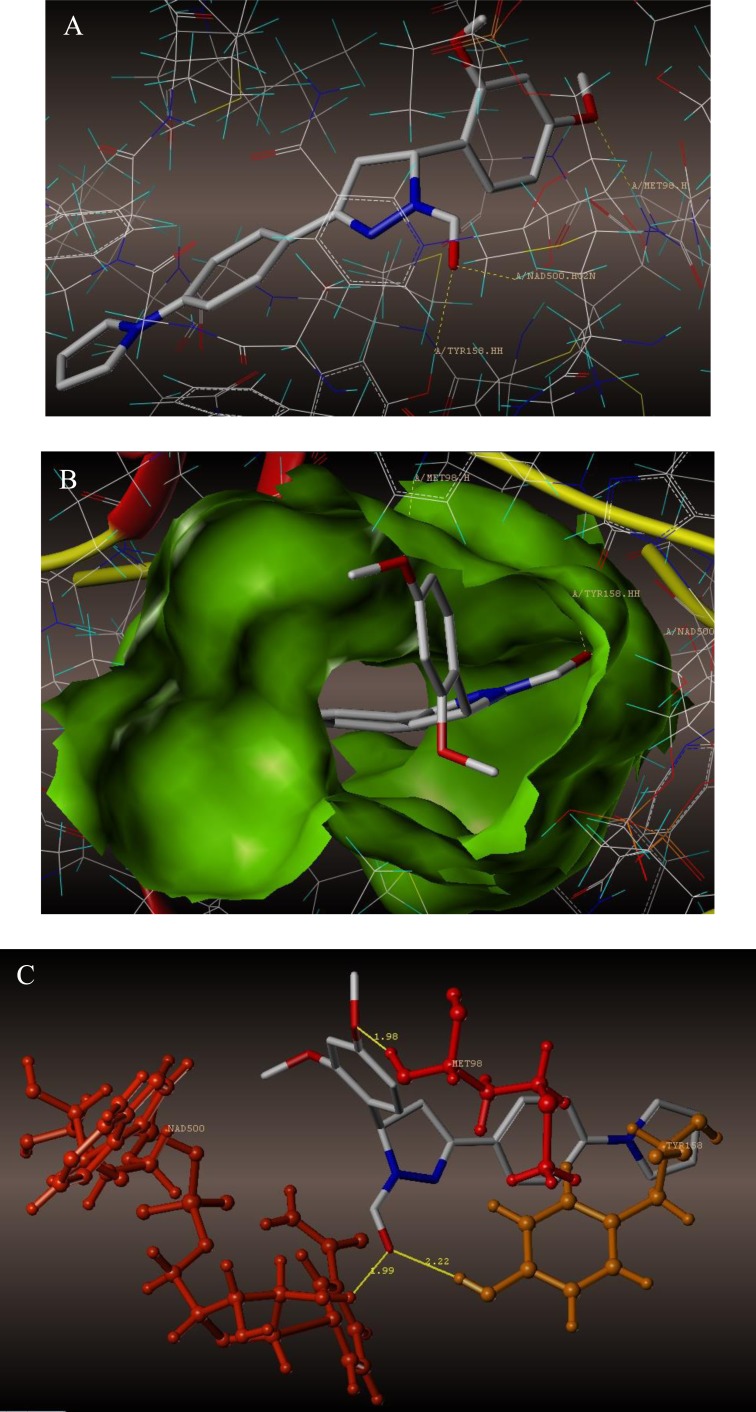
(A) Docked mode of compound **4i**; (B) Inside the proposed binding pocket of InhA; (C) 3D docked view of the compound **4i**. Binding site residues; red colored MET98 amino acid, orange colored TYR158 amino acid, red-orange colored co-factor NAD+ and the molecule is colored according to atom type.

**Fig. (6):**
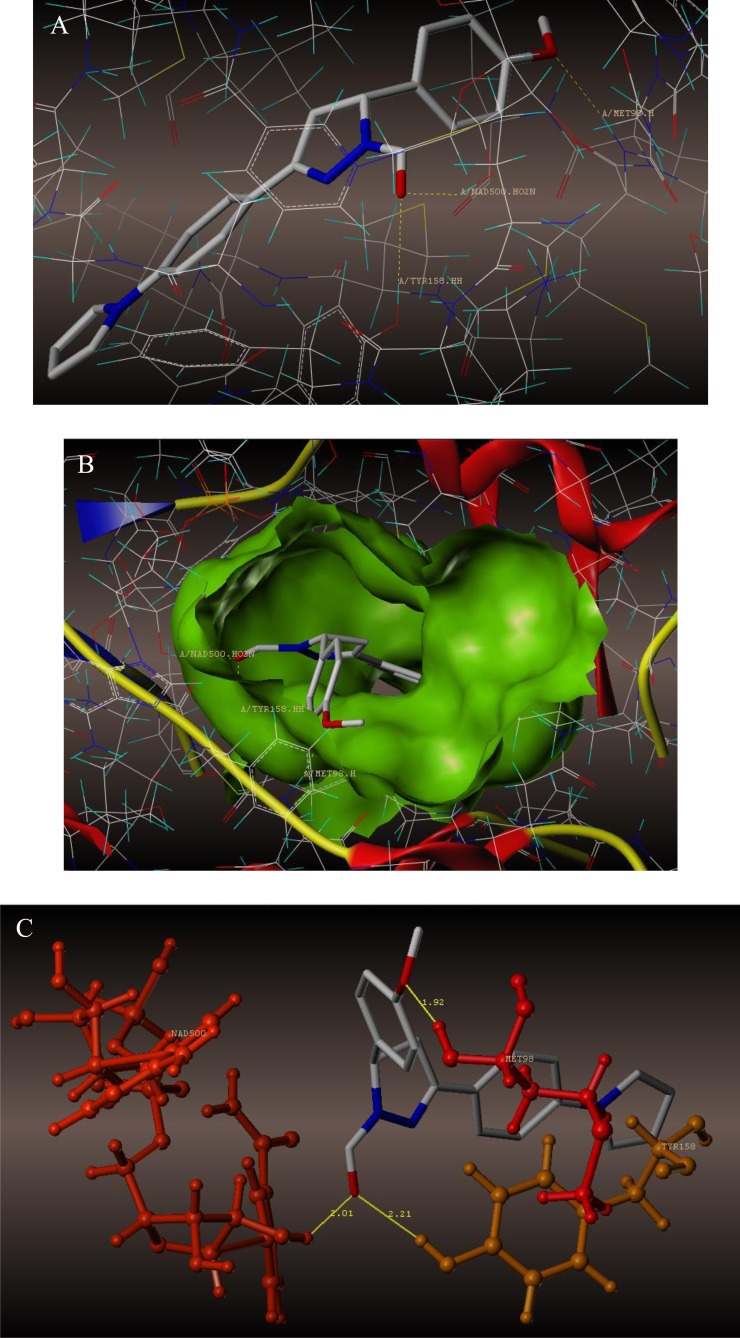
(A) Docked mode of compounds **4g**; (B) Inside the proposed binding pocket of InhA; (C) 3D docked view of the compound **4g**. Binding site residues; red colored MET98 amino acid, orange colored TYR158 amino acid, red-orange colored co-factor NAD+ and the molecule is colored according to atom type.

**Table 1:** *In vitro* evaluation of antitubercular activity.

Compounds	MIC values (µg/mL) *M. tuberculosis* H37Rv
**4a**	100
**4b**	12.5
**4c**	12.5
**4d**	25
**4e**	25
**4f**	25
**4g**	6.25
**4h**	6.25
**4i**	3.125
**4j**	12.5
**4k**	12.5
**4l**	100
**4m**	12.5
**4n**	12.5
**4o**	50
pyrazinamide	3.125
streptomycin	6.25

**Table 2:** MTT-based cytotoxicity activity of selected compounds at 100 µg/mL against human lung cancer cell line A549.

**Compounds**	**R**	**IC_50_ (**µg/mL**)**
**4b**	4-Cl	75.64
**4h**	3-OCH_3_	76.54
**4i**	2,4-diOCH_3_	73.32
**4j**	3,4-diOCH_3_	70.36
**4l**	4-CH(CH_3_)_2_	71.07
**4m**	3-Br	76.03
**4n**	3-phenoxy	72.87
**paclitaxel**	--	47.63

**IC_50_** - is half maximal inhibitory concentration**-** it is the half maximal (50%) inhibitory concentration (IC) of a substance (50% IC, or IC_50_)

**Table 3:** Surflex dock scores (kcal/mol) of pyrrolyl carbaldehyde derivatives.

Compounds	C score^a^	Crash score^b^	Polar score^c^	D score^d^	PMF score^e^	G score^f^	Chem score^g^
**4TZK ligand**	8.73	-1.39	1.18	-168.11	-49.19	-285.29	-37.47
**4a**	3.40	-1.41	0.83	-87.40	-41.73	-190.46	-27.90
**4b**	6.19	-1.37	1.69	-147.90	-47.25	-255.46	-42.47
**4c**	3.49	-0.48	1.05	-86.56	-47.61	-158.83	-29.30
**4d**	5.01	-1.94	1.92	-151.37	-51.20	-260.16	-43.90
**4e**	3.04	-0.55	1.12	-84.73	-43.52	-151.65	-29.31
**4f**	2.83	-3.07	0.12	-131.05	-33.56	-248.71	-34.59
4g*	8.12	-1.19	2.82	-152.40	-47.00	-257.44	-45.80
**4h**	6.56	-1.29	1.85	-142.70	-47.55	-244.00	-41.24
4i*	9.05	-1.14	2.80	-158.79	-44.58	-271.45	-45.43
**4j**	4.68	-0.65	0.01	-105.74	-66.28	-200.18	-29.53
**4k**	2.65	-0.92	0.03	-77.96	-45.30	-181.13	-25.78
**4l**	6.03	-2.35	2.04	-147.08	-49.99	-254.94	-44.66
4m*	6.85	-1.22	1.93	-143.05	-42.04	-249.57	-42.83
4n*	7.04	-1.31	0.78	-153.99	-43.07	-254.34	-39.23
**4o**	5.53	-0.87	0.00	-116.69	-44.22	-231.94	-32.73

* Astrisk indicates the compounds with better docking score. ^a^ CScore (Consensus Score) integrates a number of popular scoring functions for ranking the affinity of ligands bound to the active site of a receptor and reports the output of total score. ^b^ Crash-score revealing the inappropriate penetration into the binding site. Crash scores close to 0 are favourable. Negative numbers indicate penetration. ^c^ Polar indicating the contribution of polar interactions to the total score. ^d^ D-score for charge and van der Waals interactions between the protein and the ligand. ^e^ PMF-score indicating Helmholtz free energies of interactions for protein-ligand atom pairs (Potential of Mean Force, PMF). ^f^ G-score showing hydrogen bonding, complex (ligand-protein), and internal (ligand-ligand) energies. ^g^ Chem-score points for H-bonding, lipophilic contact, and rotational entropy, along with an intercept term.

**Table 4:** QSAR parameters: cLogP, molecular weight (MW), number of H bond donors and H bond acceptors value for compounds **4(a-o)**.

**Compounds**	**cLogP**	**Donor**	**Acceptor**	**Lipinski violations**	**Mol. wt**
**4a**	3.43	0	4	0	315.36
**4b**	4.14	0	4	0	349.81
**4c**	4.74	0	4	0	384.25
**4d**	4.86	0	4	0	384.25
**4e**	4.86	0	4	0	384.25
**4f**	3.93	0	4	0	329.39
**4g**	3.35	0	5	0	345.39
**4h**	3.35	0	5	0	345.39
**4i**	3.44	0	6	0	375.42
**4j**	3.09	0	6	0	375.42
**4k**	3.57	0	4	0	333.35
**4l**	4.86	0	4	0	357.44
**4m**	4.30	0	4	0	394.26
**4n**	5.53	0	5	1	407.46
**4o**	2.61	0	5	0	305.33
